# Defining post-obstructive diuresis following posterior urethral valve ablation

**DOI:** 10.3389/fped.2025.1584878

**Published:** 2025-07-04

**Authors:** Callum Lavoie, Brian Chun, Christine Do, Zoë Baker, Philippe Friedlich, Andy Y. Chang

**Affiliations:** ^1^ Division of Urology, Children’s Hospital Los Angeles, USC Institute of Urology, Keck School of Medicine of USC, Los Angeles, CA, United States; ^2^ Division of Neonatology, Children’s Hospital Los Angeles, USC Institute of Urology, Keck School of Medicine of USC, Los Angeles, CA, United States

**Keywords:** posterior urethral valves, post-obstructive diuresis, urethral valve ablation, urinary obstruction, pediatric urology

## Abstract

**Introduction:**

Posterior urethral valves (PUV) are the most common cause of congenital lower urinary tract obstruction. Patients are at risk for post-obstructive diuresis (POD) following management of this obstruction which may prolong and/or complicate their subsequent hospital course. Despite this known physiologic effect, there is minimal data to define which patients are at highest risk for POD. Our objective was to define an initial urine output threshold for neonatal post-obstructive diuresis.

**Methods:**

A retrospective chart review was conducted on patients that were admitted to our Newborn and Infant Critical Care Unit (NICCU), in a tertiary care children's hospital, between 2004 and 2019 and underwent cystoscopic valve ablation for PUV. Outcomes of interest were length of hospital stay after posterior urethral valve ablation, serum creatinine and electrolyte values, fluid intake, and urine output at 4- and 24-h post-valve ablation. Chi-squared, Fisher's exact, and *T*-tests were conducted for descriptive statistical analyses as appropriate. Logistic regression analyses were conducted with adjusted models including patient demographic and clinical data.

**Results:**

Forty patients met inclusion criteria and the mean age at time of valve ablation was 11.2 days. Pre-operatively, maximum creatinine levels (ng/dl) achieved had a median value of 0.7 (IQR: 0.5–1.5). Post-operatively, the mean urine output (mL/kg/h) at 4-h was 4.2 ± 3.7, and at 24-h was 4.5 ± 2.2. Logistic regression analyses showed that those with a post-operative 24-h UOP > 3.5 ml/kg/h had more than 5 times the odds of a prolonged hospital length of stay (LOS) > 3 days (OR: 5.50; 95% CI: 1.23–24.51).

**Discussion:**

Neonates with PUV who undergo valve ablation are at risk of POD. Our findings suggest greater urine output after ablation to be a predictor of increased hospital length of stay. Utilizing a urine output (UOP) of >3.5 mL/kg/h may serve as a starting point for defining POD after posterior urethral valve ablation.

## Introduction

Nearly one in 500 pregnancies are impacted by urinary tract congenital anomalies, with obstructive uropathy being the most common (1, 2). Lower urinary tract obstruction, specifically, is estimated to occur in 2.2 per 10,000 births. The most common lower urinary tract obstruction is posterior urethral valves (PUV), occurring in 1.4 per 10,000 births (3–5). This congenital obstructive uropathy affects male neonates and is characterized by the persistence of abnormal tissue folds or membranes in the posterior urethra, leading to urinary tract obstruction (6). Routine management consists of initial decompression with urethral catheterization followed by definitive management with valve ablation and/or vesicostomy for optimized drainage to avoid further renal damage and preserve renal function (7). Despite timely treatment and optimal management, ∼30% of patients treated for PUV in infancy develop kidney failure requiring dialysis and ultimately renal transplantation during childhood or adolescence (7–9). In the seminal paper by Warshaw et al., children with nadir creatinine values ≤0.8 mg/dl by 12 months of age had improved renal function (creatinine ≤ 1.1 mg/dl) at an average 5.8 year follow-up, compared to children with nadir creatinine values >0.8 mg/dl, who were more likely to progress to renal failure (10). Patients with a history of PUV are more prone to develop lower urinary tract dysfunction which in turn increases the risk of renal failure (11, 12).

More acutely, management of posterior urethral valves by ablation can result in post-obstructive diuresis (POD), which is an important physiologic process and clinical finding that can occur after relief of urinary obstruction. POD is well-studied in the adult population and is associated with excessive urine output and serum electrolyte disturbances which can be life-threatening without intervention. These abnormalities are theorized to stem from renal tubular injury secondary to chronic long-term obstruction, resulting in a loss of tubular concentrating ability and polyuria (13–15). Adult risk factors for POD include elevated creatinine and bicarbonate, urinary retention, heart failure, volume overload, and central nervous system depression (16, 17). In pediatric patients—and more specifically neonatal patients—these risk factors have not been clearly defined. The urine output threshold that defines POD in adults cannot be reliably extrapolated to the neonatal population due to the immaturity of the kidney's urine concentrating ability and different water body composition compared to adults (5). Without a clear definition of POD in children, an infant's risk of POD following posterior urethral valve ablation (PUVA) is usually empirically managed with fluid support and close inpatient monitoring.

This study aims to improve our understanding of POD in neonates post-PUVA by investigating the clinical course of patients at our center following valve ablations. We sought to define an initial urine output threshold for neonatal POD, which can help identify high risk patients who require closer observation, counsel families, and provide short and long-term prognoses.

## Materials and methods

### Patient selection

A retrospective chart review was conducted on patients that were admitted to our institutions' Newborn and Infant Critical Care Unit (NICCU), in a tertiary care children's hospital, between 2004 and 2019 and who underwent cystoscopic valve ablation for PUV. This included both patients born at our institution as well as patients transferred for escalation of care. Patient demographic, procedural (PUVA, catheter placement, VCUG), and clinical data (gestational age, weight, laboratory values, urine output, fluid intake) were collected, recorded, and de-identified. We excluded patients with diagnosed or identified respiratory insufficiency or pathology requiring investigation and management (at the discretion of the attending neonatologist in our chart review), patients with other diagnosed congenital abnormalities, and patients who underwent delayed valve ablation beyond 30 days of life. All patients had a catheter inserted prior to their valve ablation operation either before or after the diagnosis of PUV was confirmed on voiding cystourethrogram (VCUG). The surgical technique of valve ablation or incision was at the discretion of the operating surgeon, however, all patients had a urethral catheter left in place post-procedurally.

### Outcomes

The primary outcome of interest in this study was length of stay in hospital post-PUVA, which was selected as the clinical endpoint dictating a patient's ability to maintain hydration with only oral intake and without electrolyte abnormalities. Patients had serial evaluations in weight, physical exams, urine output and serum electrolyte labs to determine hydration status. Patients who were unable to maintain adequate hydration orally were supplemented with IV hydration at a rate lower than total UOP to minimize the diuresis effect. Secondary outcomes were urine output (ml/kg/h) at 4 and 24 h post-PUV ablation.

### Statistical analysis

A sample size of at least 40 patients was calculated to measure for statistical significance with 80% power. Chi-squared, Fisher's exact, and *T*-tests were conducted for descriptive statistical analyses as appropriate. Logistic regression analyses were conducted, with adjustments for any non-normal distribution. Adjusted models including demographic and relevant clinical data. All statistical analyses were performed using SAS (version 9.4; SAS Institute Inc., Cary, NC). Approval for the study was obtained from the Children's Hospital of Los Angeles institutional review board (CHLA-19-00297).

## Results

A total of 40 patients from 2004 to 2019 met inclusion criteria. The mean age at time of valve ablation was 11.2 days with no patients undergoing valve ablation in their first 4 days of life, and 70% (*n* = 28) of patients had a gestational age ≥37 weeks. The gestational age range was 32–41 weeks, with a mean of 37.43 ± 2.26 weeks. The median weight at birth was 3.1 kg (IQR: 2.6–3.7) compared to 3.2 kg (IQR: 2.6–3.6) at time of surgery. Vesicoureteral reflux (VUR) was identified on VCUG in 42.5% (*n* = 17) of patients. Pre-operatively, maximum creatinine levels (ng/dl) achieved had a median value of 0.7 (IQR: 0.5–1.5) and maximum blood urea nitrogen (BUN) levels (ng/dl) had a median value of 11.0 (IQR: 5.0–18.0). All pre-operative creatinine and BUN values came after day of life 4. Mean pre-operative Na and K values were 140.5 ± 3.6 and 4.7 ± 0.7, respectively, with no difference compared to post-operative values (Table 1).

**Table 1:** Distribution of characteristics of patients who underwent valve ablation for posterior urethral valves within the first 30 days of life (*n* = 40 patients).

Variable	*n* = 40 patients
Gestational age at birth, *n* (%)	
<37 weeks	12 (30.0)
≥37 weeks	28 (70.0)
Mean weight at birth, kg	3.1 ± 0.7
Mean weight at surgery, kg	3.2 ± 0.7
Mean age at surgery, days	11.2 ± 8.5
Mean 4 h post-op urine output, ml/kg/h	4.2 ± 3.7
Mean 24 h post-op urine output, ml/kg/h	4.5 ± 2.2
Total fluid intake 24 h post-op, ml/kg/h, median (IQR)	6.7 (5.4–7.5)
Total enteral fluid intake 24 h Post-Op, ml/kg/h, median (IQR)	4.2 (1.0–6.6)
Total IV fluid intake 24 h Post-Op, ml/kg/h, median (IQR)	4.2 (0.9–4.7)
Total fluid intake above maintenance needs^a^, ml/kg, median (IQR)	60.9 (28.9–80.9)
Evidence of VUR, *n* (%)	
Yes	17 (42.5)
No	23 (57.5)
Peak Creatinine, mg/dl, median (IQR)	0.7 (0.5–1.5)
Peak BUN, mg/dl, median (IQR)	11.0 (5.0–18.0)
Mean Na (mmol/L)	
Pre-Op	140.5 ± 3.6
Post-Op	140.6 ± 3.7
Change (Pre-Op to Post-Op)	−0.2 ± 3.3
Mean K (mmol/L)	
Pre-Op	4.7 ± 0.7
Post-Op	4.7 ± 0.9
Change (Pre-Op to Post-Op)	0.0 ± 0.8
Breastfed^b^, *n* (%)	
Yes	13 (34.2)
No	25 (65.8)
Length of post-operative stay, *n* (%)	
≤3 days	14 (35.0)
4–7 days	11 (27.5)
>7 days	15 (37.5)

^a^ Maintenance = 100 ml/kg/24 h.

^b^ Missing = 2.

Post-operatively, the mean urine output (UOP) (ml/kg/h) at 4-h was 4.2 ± 3.7, and at 24-h was 4.5 ± 2.2 (Table 1). The median total fluid intake over the first 24 h post-op was 6.7 (5.4–7.5) ml/kg/h, and the total intravenous fluid intake over this same period was 4.2 (0.9–4.7) ml/kg/h. For post-operative length of hospital stay (LOS), the largest proportion of study patients had an LOS >7 days (37.5%).

Logistic regression analyses showed that those with a post-operative 24-h UOP > 3.5 ml/kg/h had more than 5 times the odds of a LOS > 3 days (OR: 5.50; 95% CI: 1.23–24.51) (Table 2). Other factors significantly associated with prolonged LOS included greater patient weight (OR: 0.30; 95% CI: 0.10–0.94), increased pre-operative Cr (OR: 1.19; 95% CI: 1.01–1.40), increased BUN (OR: 1.11; 95% CI: 1.02–1.21). Interestingly, an increased pre-operative Na level was associated with lower odds of prolonged LOS (OR: 0.78; 95% CI: 0.61–0.99) (Table 2). Secondary logistic regression analyses investigating factors predictive of 4- and 24-h post-operative UOP > 3.5 ml/kg/h showed no association with peak creatinine, peak BUN, sodium, weight, gestational age, fluid intake, and breastfeeding status (Table 3).

**Table 2:** Results of logistic regression analyses assessing factors associated with increased post-operative hospital stay and need for post-operative intravenous (IV) fluids.

Variable	Outcome: stay > 3 days	
	OR (95% CI)	*p*-value
4 h Urine output (per 1 ml/kg/h increase)	1.12 (0.91–1.37)	0.25
24 h Urine output (per 1 ml/kg/h increase)	1.51 (1.03–2.21)	0.02*
UOP > 3.0 ml/kg/h	3.07 (0.58–16.31)	0.69
UOP > 3.5 ml/kg/h	5.50 (1.23–24.51)	0.02*
UOP > 4.0 ml/kg/h	6.00 (1.44–24.92)	0.01*
UOP > 4.5 ml/kg/h	4.00 (1.00–16.27)	0.04*
UOP > 5.0 ml/kg/h	6.00 (1.12–32.28)	0.02*
Gestational age	0.78 (0.57–1.09)	0.13
Age at surgery	0.99 (0.93–1.07)	0.99
Weight at surgery (kg)	0.30 (0.10–0.94)	0.03*
Change in weight from birth to surgery (per g/day)	1.03 (1.00–1.06)	0.03*
Peak creatinine (mg/dl; per 0.1 increase)	1.19 (1.01–1.40)	0.004**
Peak BUN (mg/dl)	1.11 (1.02–1.21)	0.001**
Na (mmol/L)		
Pre-Op	0.78 (0.61–0.99)	0.02*
Post-Op	0.82 (0.66–1.02)	0.06
Change (Pre-Op to Post-Op)	1.00 (0.81–1.23)	0.99
K (mmol/L)		
Pre-Op	0.63 (0.22–1.80)	0.38
Post-Op	0.96 (0.44–2.13)	0.93
Change (Pre-Op to Post-Op)	1.42 (0.55–3.64)	0.46
VUR	1.83 (0.45–7.41)	0.39
Fluid Intake > 100 ml/kg/24 h	0.67 (0.13–3.52)	0.63

* *p* < 0.05.

** *p* < 0.005.

**Table 3:** Results of logistic regression analyses for association of factors with post-op 4- and 24-h urine output.

Variable	4 h UOP		24 h UOP	
	≥3.5 ml/kg/h		≥3.5 ml/kg/h	
	OR (CI)	*p*-value	OR (CI)	*p*-value
Gestational age (weeks)	1.00 (0.59–1.72)	0.9932	0.87 (0.51–1.50)	0.6122
Age at surgery (days)	1.01 (0.88–1.15)	0.9216	1.16 (0.94–1.42)	0.1697
Weight at surgery (kg)	1.09 (0.20–6.15)	0.9187	1.52 (0.25–9.18)	0.6481
Change in weight from birth to surgery (per g/day)	1.03 (0.98–1.07)	0.2349	1.02 (0.98–1.07)	0.2630
Peak creatinine (mg/dl; per 0.1 increase)	0.71 (0.25–2.00)	0.5108	1.20 (0.50–2.92)	0.6820
Peak BUN (mg/dl)	0.93 (0.81–1.07)	0.3374	0.98 (0.90–1.07)	0.6085
Na (mmol/L)				
Pre-Op	1.02 (0.76–1.37)	0.8897	1.10 (0.83–1.46)	0.4951
Post-Op	1.28 (0.79–2.09)	0.3166	0.97 (0.70–1.34)	0.8287
Change (Pre-Op to Post-Op)	1.70 (0.73–3.96)	0.2158	1.00 (0.73–1.36)	0.9843
K (mmol/L)				
Pre-Op	0.75 (0.14–3.92)	0.7307	2.48 (0.45–13.77)	0.3002
Post-Op	0.04 (0.00–0.65)	0.0244*	0.55 (0.14–2.15)	0.3860
Change (Pre-Op to Post-Op)	0.00 (0.00–0.64)	0.0338*	0.47 (0.13–1.68)	0.2445
VUR	0.20 (0.03–1.34)	0.0967	1.61 (0.20–12.69)	0.6541
Fluid Intake > 100 ml/kg/24 h	1.79 (0.10–30.82)	0.6896	0.09 (0.00–2.17)	0.1361
Breastfeeding	1.36 (0.20–9.12)	0.7536	0.95 (0.15–6.13)	0.9579

* *p* < 0.05.

## Discussion

Post-obstructive diuresis (POD) is a clinical entity that is well-defined for adults with known risk factors, standardized management strategies, and prognoses. However, there is little data on how this process pertains to pediatrics and neonatal patients, particularly in the setting of posterior urethral valves (PUV). Our study evaluated neonatal patients with an average age of 11.2 days at the time of their posterior urethral valve ablation (PUVA) procedure and found high urine output to be a predictor of prolonged hospitalization, which may indicate a need for supplemental intravenous fluid resuscitation. Specifically, patients with a urine output greater than 3.5 ml/kg/h at 24 h post-operatively were 5.5 times more likely to stay in the hospital for more than 3 days post-operatively.

Bermeo et al. investigated risk factors for POD in pediatric patients post-pyeloplasty and used a threshold value for POD of urine output >5 cc/h for >2 h post-operatively (18) and found an association with younger age, lower weight, and previous renal tubular acidosis. Although these factors were found to be associated with their definition of POD, they did not allow the data to define the threshold of POD with a regression model. Additionally, their model is not compatible with the traditional definition of POD as this evaluated a unilateral obstructed system. Notably, they did not identify any electrolyte derangements in the POD cohort compared to their control group. Sartorius et al. similarly investigated the incidence of POD after PUVA in neonatal patients. Using a urine output threshold of 6 ml/kg/h in the immediate 24-h after initial relief of obstruction (via urethral catheter, suprapubic tube, or surgical ablation), 15/40 of their sample met the criteria to be defined with POD, and these patients were more likely to have electrolyte abnormalities such as elevated serum creatinine and urea, and hyponatremia. These findings were representative of a heterogeneous cohort which included patients who underwent antenatal vesico-amniotic shunting, postnatal urinary diversion, and those with other significant comorbidities including respiratory insufficiency. Additionally, there was heterogeneity in the initial management of obstruction with 42.5% having a urethral catheter placed and 10% receiving endoscopic ablation (19). By comparison our study represents a more homogeneous, clinically stable patient cohort which we believe reduces the confounding effect of multiple comorbidities. Indeed, 35% of our patient cohort was discharged from the hospital in under 3 days post-operatively, and 27.5% were discharged from the hospital between 4 and 7 days post-operatively.

Our study has notable limitations. Firstly, it is a retrospective, single institution study with a limited dataset given the rarity of PUV, although our data reflects the cumulative experience of a large metropolitan tertiary pediatric care center spanning 15 years. Prenatal variables such as gestation age at diagnosis, oligohydramnios, degree and laterality of hydronephrosis were not available in all sampled patients. This study cannot be generalized to all patients with PUV nor any other causes of urinary obstruction as these patients often present with other confounding comorbidities such as respiratory insufficiency requiring mechanical ventilation; our study selectively excluded these more complex patients with the aim to specifically investigate post-obstructive diuresis secondary to posterior urethral valves. Our institution is a large referral center with many patients who are initially managed at an outside institution, then subsequently transferred for PUVA, therefore, data such as initial serum laboratory of urine output at the time of catheter placement is limited. We acknowledge that other clinical and social factors may influence a patient's hospital length of stay, but we believe this effect is minimized by excluding medically complex patients. Furthermore, although no pre-operative creatinine and BUN values included in our analysis were drawn within the first 4 days of life, it is possible that some patients had not fully cleared their maternal serum levels by this time and these values may be artificially elevated, especially if optimal drainage had not yet been achieved. Additionally, although these patients were able to undergo earlier treatment because they were medically stable at an average age of 11.2 days, it is important to recognize that a feeding tube or catheter would have been inserted into their bladder pre-operatively either at birth or upon confirmation with VCUG, and some diuresis may likely have already occurred prior to their procedure, however, similar studies suggest that the diuretic effect appears to last for several days after decompression (19). Although a catheter should be placed as a temporizing measure until surgical ablation, this can be particularly challenging in PUV patients due to anatomic differences such as a dilated posterior urethra and bladder neck hypertrophy (20). Even with catheter placement, the relief of obstruction may be suboptimal due to limitations in the lumen size of feeding tubes, frequent dislodgement, tenting of the bladder, and ureteral orifice obstruction if a foley balloon is deployed (20). Another consideration is that our study includes a subset of patients who were catheterized prior to transfer to our institution for PUVA, however we believe that time of PUVA can serve as a useful objective measure for relief of obstruction because of the limitations of catheter-only decompression and because it is an easily referenced start point in patients who are transferred from outside facilities with an unknown catheter dwell period.

Our analysis illustrates an association between greater post-operative UOP with prolonged LOS while controlling for both intravenous and enteral fluid intake. Electrolyte abnormalities typically manifest as a hallmark feature of POD and our data trended towards this finding, however, we found no statistically significant differences, likely due to a limited sample size of a rare clinical entity. Despite this, the clinical significance of UOP impacting LOS suggests that diuresis continues to occur in these neonatal patients with immature renal function after definitive valve ablation. Our findings suggest urine output greater than 3.5 ml/kg/h at 24 h post-operatively as an initial threshold for high-risk patients who require close monitoring and potential IV fluid supplementation. Future work in the form of prospective, multi-center trials is needed to confirm this parameter and determine associations with long term renal function after discharge.

## Conclusion

Neonates with PUV who undergo valve ablation and urinary tract decompression are at risk of POD. Our study shows greater UOP to be a predictor of increased hospital LOS and utilizing a UOP of >3.5 ml/kg/h may be a useful starting point for defining POD after posterior urethral valve ablation.

## Data Availability

The raw data supporting the conclusions of this article will be made available by the authors, without undue reservation.

## References

[1] Lissauer D Morris RK Kilby MD. Fetal lower urinary tract obstruction. Semin Fetal Neonatal Med. (2007) 12:464–70. 10.1016/j.siny.2007.06.005 17761463

[2] Ruano R. Fetal surgery for severe lower urinary tract obstruction. Prenat Diagn. (2011) 31:667–74. 10.1002/pd.2736 21413041

[3] Anumba DO Scott JE Plant ND Robson SC. Diagnosis and outcome of fetal lower urinary tract obstruction in the northern region of England. Prenat Diagn. (2005) 25(1):7–13. 10.1002/pd.1074 15662711

[4] Alsaywid BS Mohammed AF Jbril SM Bahashwan M Mukharesh L Al Khashan M. Renal outcome among children with posterior urethral valve: when to worry? Urol Ann. (2021) 13(1):30–5. 10.4103/UA.UA_112_19 33897161 PMC8052897

[5] Chawla D Agarwal R Deorari AK Paul VK. Fluid and electrolyte management in term and preterm neonates. Indian J Pediatr. (2008) 75:255–9. 10.1007/s12098-008-0055-0 18376094

[6] Young HH Frontz WA Baldwin JC. Congenital obstruction of the posterior urethra. J Urol. 1919;3:289-365. J Urol. (2002) 167(1):265–7; discussion 268. 10.1016/S0022-5347(05)65444-4 11743334

[7] Hodges SJ Patel B McLorie G Atala A. Posterior urethral valves. Sci World J. (2009) 9:1119–26. 10.1100/tsw.2009.127 PMC5823193 19838598

[8] Heikkila J Holmberg C Kyllonen L Rintala R Taskinen S. Long-term risk of end stage renal disease in patients with posterior urethral valves. J Urol. (2011) 186:2392–6. 10.1016/j.juro.2011.07.109 22014822

[9] Capone V Persico N Berrettini A Decramer S De Marco EA De Palma D Definition, diagnosis and management of fetal lower urinary tract obstruction: consensus of the ERKNet CAKUT-obstructive uropathy work group. Nat Rev Urol. (2022) 19(5):295–303. 10.1038/s41585-022-00563-8 35136187

[10] Warshaw BL Hymes LC Trulock TS Woodard JR. Prognostic features in infants with obstructive uropathy due to posterior urethral valves. J Urol. (1985) 133(2):240–3. 10.1016/S0022-5347(17)48899-9 3968741

[11] DeFoor W Clark C Jackson E Reddy P Minevich E Sheldon C. Risk factors for end stage renal disease in children with posterior urethral valves. J Urol. (2008) 180(4 Suppl):1705–8; discussion 1708. 10.1016/j.juro.2008.03.090 18708224

[12] Ansari MS Gulia A Srivastava A Kapoor R. Risk factors for progression to end-stage renal disease in children with posterior urethral valves. J Pediatr Urol. (2010) 6(3):261–4. 10.1016/j.jpurol.2009.09.001 19833558

[13] Li C Klein JD Wang W Knepper MA Nielsen S Sands JM Altered expression of urea transporters in response to ureteral obstruction. Am J Physiol Renal Physiol. (2004) 286(6):F1154–62. 10.1152/ajprenal.00453.2003 14982816

[14] Nguyen HT Hsieh MH Gaborro A Tinloy B Phillips C Adam RM. JNK/SAPK and p38 SAPK-2 mediate mechanical stretch-induced apoptosis via caspase-3 and -9 in NRK-52E renal epithelial cells. Nephron Exp Nephrol. (2005) 102:e49–61. 10.1159/000088401 16179830

[15] Dinneen MD Duffy PG Barratt TM Ransley PG. Persistent polyuria after posterior urethral valves. Br J Urol. (1995) 75:236–40. 10.1111/j.1464-410X.1995.tb07318.x 7850332

[16] Gonzalez CM. Pathophysiology, diagnosis, and treatment of the postobstructive diuresis. In: McVary KT, editor. Management of Benign Prostatic Hypertrophy. New York City, NY: Humana Press (2004); Current Clinical Urology.

[17] Hamdi A Hajage D Van Glabeke E Belenfant X Vincent F Gonzalez F Severe post-renal acute kidney injury, post-obstructive diuresis and renal recovery. BJU Int. (2012) 110:E1027–34. 10.1111/j.1464-410X.2012.11193.x 22583774

[18] Pedraza Bermeo AM Ortiz Zableh AM Castillo M Pérez Niño JF. Risk factors for postobstructive diuresis in pediatric patients with ureteropelvic junction obstruction, following open pyeloplasty in three high complexity institutions. J Pediatr Urol. (2018) 14(3):260.e1–e4. 10.1016/j.jpurol.2018.01.017 29501380

[19] Sartorius V Giuseppi A Iacobelli S Leroy-Terquem E Vinit N Heidet L Post-obstructive diuresis after posterior urethral valve treatment in neonates: a retrospective cohort study. Pediatr Nephrol. (2024) 39(2):505–11. 10.1007/s00467-023-06100-y 37656311

[20] Penna FJ Bowlin P Alyami F Bägli DJ Koyle MA Lorenzo AJ. Novel strategy for temporary decompression of the lower urinary tract in neonates using a ureteral stent. J Urol. (2015) 194(4):1086–90. 10.1016/j.juro.2015.04.102 25963187

